# New scientific analyses reveal mixing of copper sources in the early Iron Age metal production at Ili, western China

**DOI:** 10.1111/arcm.12770

**Published:** 2022-03-15

**Authors:** Cheng Liu, Ruiliang Liu, Siying Zhu, Jie Wu, A. Mark Pollard, Jianfeng Cui, Jianyi Tong, Limin Huan, Yiu‐Kang Hsu

**Affiliations:** ^1^ School of Cultural Heritage Northwest University Xi'an China; ^2^ The Department of Asia British Museum London UK; ^3^ School of Archaeology University of Oxford Oxford UK; ^4^ Emperor Qin Shihuang's Mausoleum Site Museum Xi'an China; ^5^ Xinjiang Uygur Autonomous Region Museum Urumqi China; ^6^ School of Archaeology and Museology Peking University Beijing China; ^7^ Deutsches Bergbau‐Museum Bochum Bochum Germany

**Keywords:** cauldrons, early metallurgy, Lead isotopes, mixing and recycling, Western Xinjiang

## Abstract

The crucial role that Xinjiang played in cultural communication across the Eurasian steppe in prehistory is evidenced by the large number of copper‐based objects that represent the early metallurgical technologies found across this region. Our research adds new chemical and isotopic analyses of 44 copper‐based objects dated to the early Iron Age of Ili in Xinjiang, western China. As noted in a number of publications, tin bronze and arsenic copper/bronze were the dominant alloying types across Xinjiang during the second and first millennium BC, whereas some specific types of objects such as cauldrons are often made from pure copper. The western Tianshan Mountain, including the well‐known mining site Nulasai, is the most likely copper source for the Ili metalworking. Meanwhile, a combination of lead isotopes, lead concentrations and trace elemental data reveals new evidence for the mixing and recycling of different sources of copper.

## INTRODUCTION

The north and north western bounds of ancient Xinjiang were steppe and semisteppe areas with a landscape favouring a pastoral lifestyle as opposed to the agricultural lifestyle that the Central Plains and Southern China supported. It is likely that the differences in geography, climate, and lifestyles explain the differing way in which copper and copper alloys were used (Hsu et al., 2016; Mei, 2003; Pollard et al., 2017). Many tombs discovered in the Central Plains from the Bronze Age Shang (ca. 1,500–1,050 BC) and Western Zhou (ca. 1,050–771 BC) dynasties have been found to contain an extraordinary quantity of metal sometimes in excess of hundreds and even thousands of kilograms (e.g., the Late Shang Fu Hao tomb with ca. 1.6 ton of bronzes). Similar examples have been found along the Yangtze River, at Panlongcheng, Sanxingdui, and Dayangzhou (Bagley, 1988; Liu et al., 2019). The most distinct object type in these tombs is ritual vessels cast from complex clay moulds. Whereas in Xinjiang, as is typical of the steppe region, the primary examples of metal objects are small objects including tools, weapons, and personal ornaments (Rawson, 2017). Here, the largest quantity of metal revicvered from cemetery was at Tianshanbeilu and was just over 20 kg. This contrast opens speculation as to the different models of metal production.

**FIGURE 1:**
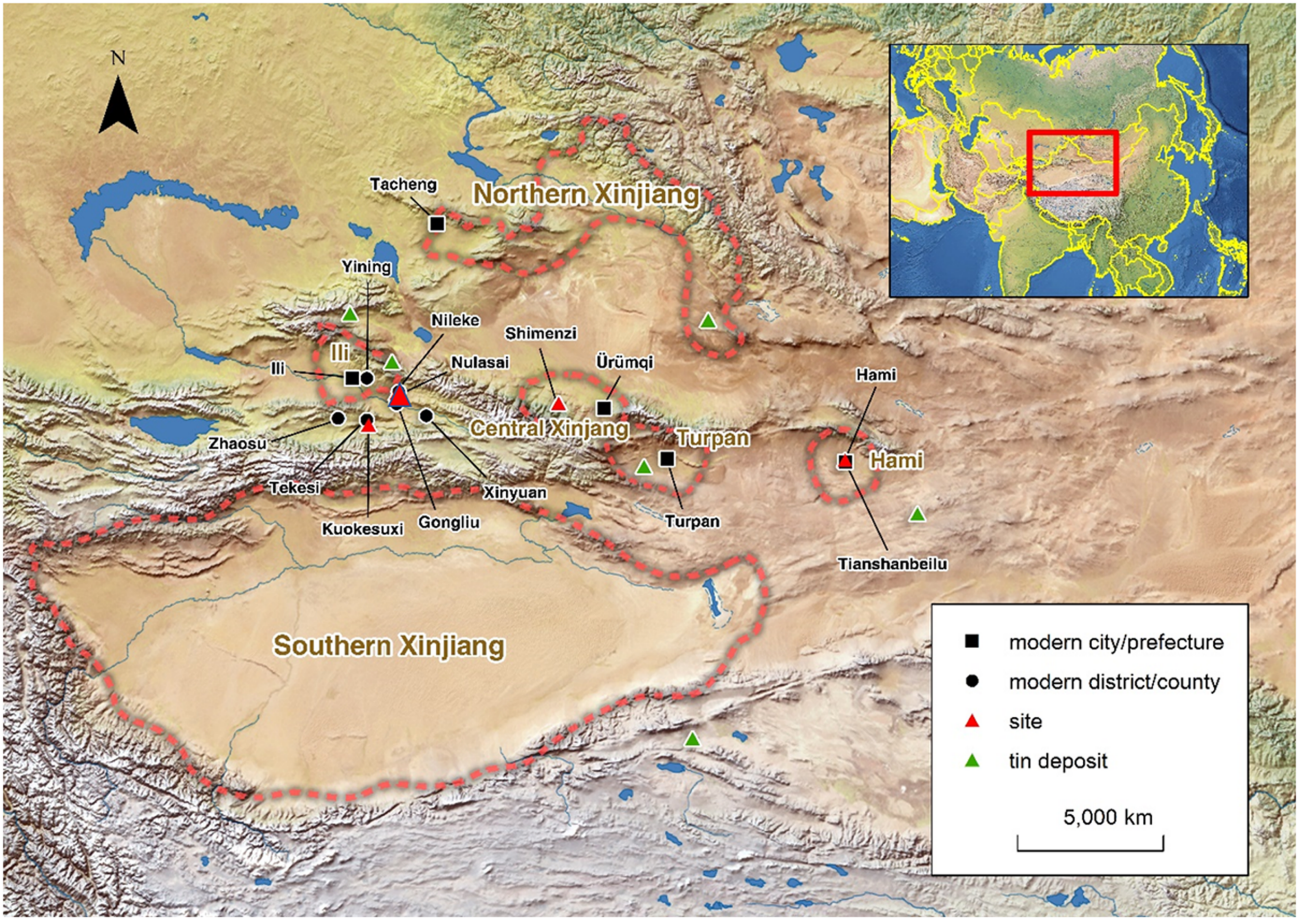
Locations of the Ili region and geographical division in the paper Notes: red dotted lines represent the main regions of Xinjiang discussed in the text [Color figure can be viewed at wileyonlinelibrary.com]

Ili's location on what is now the western border of modern China made it key to cultural communication along the ancient Silk Road (Figure 1). With the Borohoro Mountain in the north and the Tianshan Mountain in the South, the Ili River valley forms a fertile green region suitable for both agriculture and animal herding. It opens a crucial route for interaction across the entire Xinjiang region along the northern edge of the Tianshan Mountain. Its north access includes the Dzungaria Basin and the Altai Mountains, and to the northwest is the Lake Balkhash and Eastern Kazakhstan, both of which can conveniently incorporate Ili into the vast Eurasian steppe. On the southern border are routes through the Tianshan mountains linking Ili with the northern boundary of the Tarim Basin, the Taklamakan dessert and routes heading southwest towards the Inner Asian Mountain Corridor (Frachetti, 2013), corresponding with modern day Kyrgyzstan, Tajikistan, and even Afghanistan where rich sources of tin were found and extracted during the late second millennium BC. This geography was crucial to Ili's development as a hub for communication and commerce between different groups of people.

Hundreds of copper‐based objects dated to the Bronze Age (ca. 2000–1,000 BC) and early Iron Age (ca. 1,000–300 BC) have been discovered in the Ili region, mostly comprising small tools (e.g., chisels, knives or axes), ornaments (e.g., earrings, bracelets), mirrors, or weapons (e.g., arrow heads). In contrast, as one of the most typical steppe‐style artefacts, cauldrons (Fu) present a unique type of cooking vessels that were made in much larger sizes and often encountered among pastoralist societies across the entire Eurasia steppe from western China to eastern Europe during the first millennium BC (Érdy, 1995). Many of the local objects around the Ili region are believed to be associated with the Seima‐Turbino phenomenon and, most importantly, the Andronovo culture/complex, originally derived from the central Eurasian steppe (Kuz'mina, 2007).

The rich diversity of local archaeological cultures and copper‐based objects discovered from Xinjiang have attracted great scholarly attention in recent decades. An increasing number of publications have been focused on the provenance of the metal resources (e.g., Liu et al., 2020), mining and smelting (Li & Mei, 2001), alloying technologies (e.g., Mei, 2003; Qian, 2006), and their broader connection with Eurasian Steppe (e.g., Hsu, 2016). Based on the lead isotopic comparison with copper ores in the local mines of western Xinjiang, the most recent research by Wang et al. (2018) suggests that the copper‐based objects dated to the Bronze Age were imported from external sources, whereas those of the early Iron Age were more likely produced with metal from local sources such as Nulasai. However, we argue that provenance studies targeted on any Xinjiang metal group should be placed in a wider geological and social context, primarily because of the highly mobile lifestyle favoured by local people that leads to higher chance of long‐distance movement of metal objects as well as mixing and recycling. Very few scientific approaches could provide direct evidence for identification of mixing and recycling of ancient metal (see papers in this Special Issue, e.g., Berger et al., Wood, 2022). The most effective method so far is still combination of lead isotopic ratios, trace elements, and alloying composition (particularly lead concentration, Pollard & Bray, 2015). Although archaeometallurgical research in Xinjiang has been carried out for decades, to our knowledge, except the most recent work on the copper‐based objects recovered from Tianshanbeilu in eastern Xinjiang (Liu et al., 2020), there still remains a significant lack of complete dataset that covers all these types of analyses, allowing scholars to examine their potential provenances as well as mixing and recycling. This paper presents a new group of chemical and lead isotope data for the early Iron Age copper‐based objects in Ili. Along with a legacy database of ores and objects collated from the current literature, the new data enable a wider comparative study in order to explore the flow of metal resources and technologies centred on western Xinjiang. It highlights spatial and chronological patterns of alloying practice and movement of metal across different regions in prehistoric Xinjiang. The study demonstrates that the local management strategy of metal in early Iron Age Ili involved not only multiple copper sources but also the mixing and recycling of different types of copper.

## MATERIALS AND METHODS

### Sample information

This study analysed 44 objects from the Ili region and one copper ore from the Nulasai mine.

**FIGURE 2:**
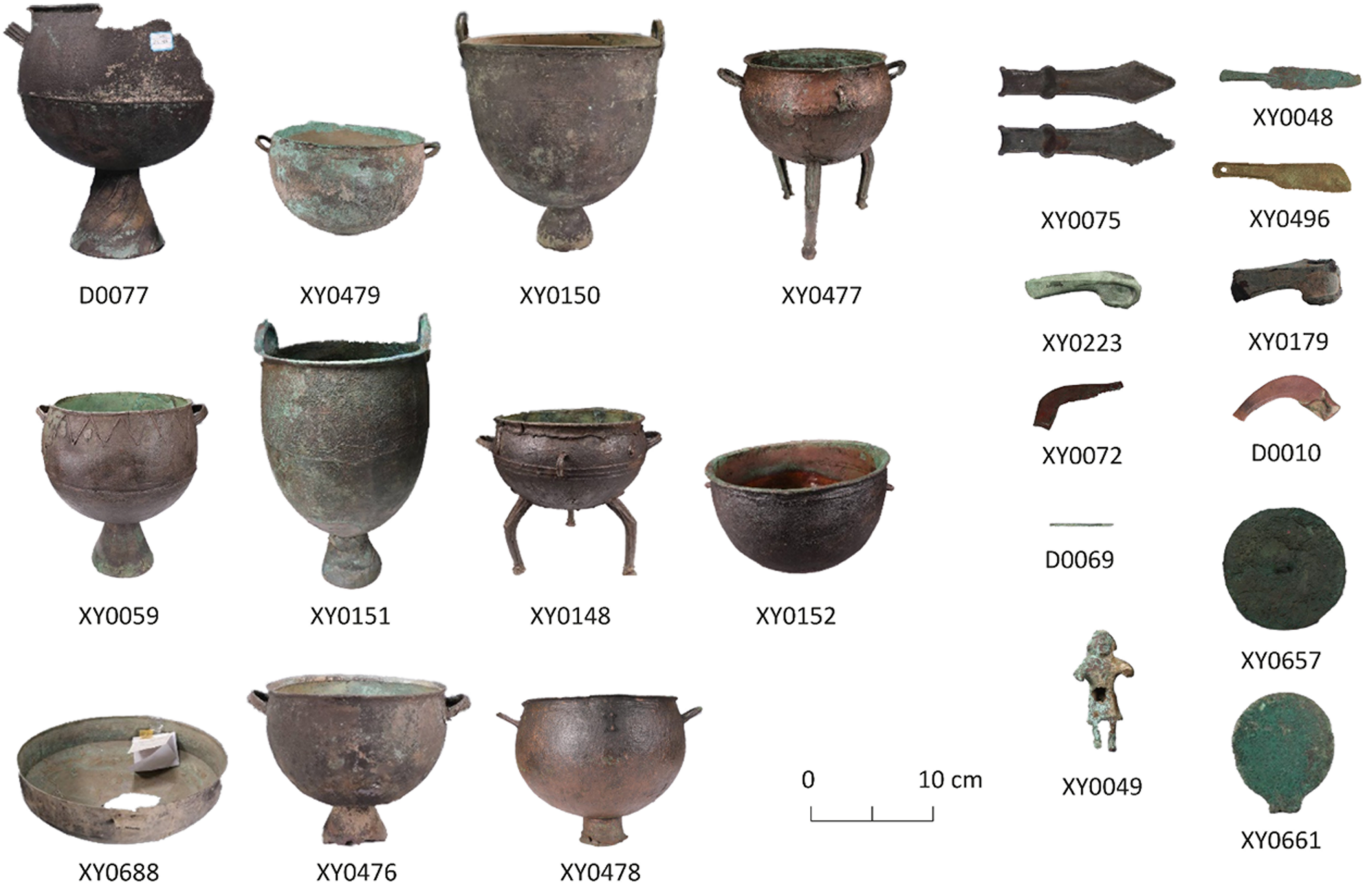
Object photos of some analysed samples in this research [Color figure can be viewed at wileyonlinelibrary.com]

The overall assemblage is typologically representative as it consists of most of the copper objects found in prehistoric Xinjiang (Table 1, Figure 2). With the exception of the mirror from the Kuokesuxi tomb II (Archaeological Institute of Xinjiang, 2012) and eight objects from the Qiabuqihai Reservoir Cemetery (Archaeological Institute of Xinjiang and China, 2006), any remaining objects are stray finds in Ili, and therefore can only be typologically dated to the early Iron Age (except the mirror from Kuokesuxi, which can be dated to end of the second millennium BC by the tomb burials and the two socketed axes related to the Andronovo culture ca. 2000–1,500 BC). Metallographic and SEM‐EDS analysis were carried out to investigate the manufacturing technology and alloying pattern. We also undertook trace elemental and lead isotopic analysis in order to characterize the underlying flow of the raw metal. A number of objects were too corroded to give meaningful results and so were excluded from the discussion below.

**TABLE 1:** Sample description and analytical methods in this study

Sample number	Object	Location	Analytical method	Sampling position
D0109	Knife	Tekesi	SEM‐EDS ICP‐MS	Blade
D0014	Arrowhead	Tekesi	SEM‐EDS ICP‐MS	Bottom
D0347	Mirror	Kuokesuxi tomb II	SEM‐EDS ICP‐MS	Edge
D0280	Hairpin	Qiabuqihai reservoir cemetery	SEM‐EDS ICP‐AES ICP‐MS	Top
D0012	Chisel	Tekesi	SEM‐EDS	Top
D0008	Spearhead	Tekesi	SEM‐EDS ICP‐MS	Top
D0073	Bowl	Qiabuqihai reservoir cemetery	SEM‐EDS ICP‐MS	Bottom
D0075	Knife	Tekesi	SEM‐EDS MC‐ICP‐MS	Blade
D0080	Bowl	Qiabuqihai reservoir cemetery	SEM‐EDS MC‐ICP‐MS	Bottom
D0111	Belt buckle	Tekesi	SEM‐EDS MC‐ICP‐MS	Edge
D0287–1	Arrowhead	Qiabuqihai reservoir cemetery	SEM‐EDS MC‐ICP‐MS	Bottom
D0287–2	Arrowhead	Qiabuqihai reservoir cemetery	MC‐ICP‐MS	Bottom
D0287–3	Arrowhead	Qiabuqihai reservoir cemetery	MC‐ICP‐MS	Bottom
D0287–4	Arrowhead	Qiabuqihai reservoir cemetery	MC‐ICP‐MS	Bottom
D0287–5	Arrowhead	Qiabuqihai reservoir cemetery	MC‐ICP‐MS	Bottom
D0432	Knife	Tekesi	SEM‐EDS MC‐ICP‐MS	Blade
D0010	Cauldron	Zhaosu	SEM‐EDS ICP‐AES MC‐ICP‐MS	Bottom
D0077	Cauldron	Zhaosu	SEM‐EDS MC‐ICP‐MS	Bottom edge
XY0657	Mirror	Nikele	SEM‐EDS MC‐ICP‐MS	Edge
XY0496	Knife	Tekesi	SEM‐EDS ICP‐AES MC‐ICP‐MS	Blade
D0083	Copper stick	Tekesi	MC‐ICP‐MS	Tail
D0078	Earring	Tekesi	MC‐ICP‐MS	Tail
XY0223	Socketed axe	Zhaosu	MC‐ICP‐MS	Blade
XY0048	Spearhead	Tekesi	MC‐ICP‐MS	Blade
XY0082	Plaque	Gongliu	MC‐ICP‐MS	Surface
XY0226	Sickle	Zhaosu	MC‐ICP‐MS	Blade
XY0661	Mirror	Nikele	MC‐ICP‐MS	Edge
XY0075	Spearhead	Yining	MC‐ICP‐MS	Edge
XY0179	Shaft‐hole axe	Gongliu	MC‐ICP‐MS	Surface
XY0479	Cauldron	Tekesi	MC‐ICP‐MS	Rim
XY0150	Cauldron	Tekesi	ICP‐AES MC‐ICP‐MS	Ring foot
XY0477	Cauldron	Zhaosu	ICP‐AES	Rim
XY0059	Cauldron	Xinyuan	ICP‐AES MC‐ICP‐MS	Ring foot
XY0151	Cauldron	Xinyuan	ICP‐AES MC‐ICP‐MS	Ring foot
XY0072	Sickle	Xinyuan	ICP‐AES	Blade
XY0156	Plate	Gongliu	ICP‐AES MC‐ICP‐MS	Bottom
XY0049	Figurine	Gongliu	ICP‐AES MC‐ICP‐MS	Back
XY0148	Cauldron	Nikele	MC‐ICP‐MS	Rim
XY0152	Cauldron	Zhaosu	SEM‐EDS ICP‐AES	Rim
XY0688	Pot	Ili	SEM‐EDS ICP‐AES MC‐ICP‐MS	Broken part in the bottom
XY0476	Cauldron	Tekesi	SEM‐EDS ICP‐AES MC‐ICP‐MS	Bottom edge
D0069	Hairpin	Tekesi	ICP‐AES MC‐ICP‐MS	Top
D0010	Sickle	Tekesi	ICP‐AES	Blade
XY0478	Cauldron	Tekesi	ICP‐AES	Bottom
——	Copper ore	Nulasai	MC‐ICP‐MS	Edge of the ore

### Analytical methods

Metallography: Samples were embedded in resin blocks of 5 cm in diameter. After grinding, polishing, and etching with solution of ferric chloride (120 mL ethanol + 30 mL hydrochloric acid + 10 g ferric chloride), six objects showed clear metallographic structures for the study of the manufacture technologies.

Alloying elements: The alloying compositional analysis was conducted in the Conservation Laboratory of Shaanxi Provincial Institute using scanning electron microscopy and energy dispersive spectrometry (SEM: ZEISS EVO MA25; EDS: X‐Max 20 of Oxford UK). All of the samples were carbon coated and measured under high vacuum, with the parameters of 20 KeV, live time 120 s (dead time around 20–30%), and calibrated using the default standardless mode. The minimum detectable limit for the majority of elements (Sn, Fe, Co, Ag, Sb, Ni) was estimated to be around 0.1% (As, Pb, Bi around 0.2%). More details of the operational process can be found in (Liu, 2016). A series of pilot analyses were conducted before the main analytical sequence in order to assess the degree of corrosion, as the corrosion of metal is a complex process that changes the different elemental concentrations dramatically. This is particularly true for small steppe‐styled objects as their size means that sometimes little sound metal can be found under SEM. In this case, the microscopic images (metallography, secondary electron, or back scattered images), along with the quantity of oxygen, sulphur, and chlorine, as well as good total of analysis (98–102%), gave important indicators of the sample's condition and the quality of the data.

Trace elements and lead isotopes: Two aliquots of the same sample were used for trace elemental and lead isotope analysis. Elemental analysis was carried out using inductively coupled plasma atomic emission spectroscopy (ICP‐AES) in the School of Archaeology and Museology, Peking University. The first stage was the removal of any corrosion, then the samples were dissolved in aqua regia, diluted to 100 mL with deionised H_2_O. A Leeman Labs Prodigy ICP‐AES was used to measure elemental compositions. The analytical conditions were RF power of 1.1 kW, argon plasma gas flow rate of 20 L/min, and nebuliser gas at 20 MPa. The detection limit was 0.1–0.01 ppm.

The lead isotopic measurements were carried out at the laboratory of Earth and Space Sciences at Peking University, using VG elemental multi‐collector‐inductively coupled plasma mass spectrometer (MC‐ICP‐MS, Nu Plasma 2). Each time it was calibrated by measuring the internationally agreed standards NBS 981 to minimize the standard deviation of the measurements. About 50 mg of each sample was dissolved in a nitric acid solution after washing and drying. Lead was then separated electrochemically by deposition onto a platinum electrode under weak nitric acid conditions. The lead on the platinum electrode was then dissolved using a few drops of 2% nitric acid into a polyethylene bottle. The purified lead solution was measured by inductively coupled plasma atomic emission spectroscopy (ICP‐AES) and then diluted by adding deionized water to 400–1,000 ppb. The international standard Thallium SRM997 was also added as internal calibration. The detailed analytical procedure follows Niederschlag et al. (2003). All of the samples were subsequently moved to MC‐ICP‐MS for isotopic measurements, and the final results are shown in Table 4.

## RESULTS

Because a few objects are too small or corroded, we were unable to take adequate amount of sample for all types of analysis. Only seven objects show clear metallographic structures, indicating that casting and hot working were the two main manufacturing techniques used for these artefacts. However, one knife (D0432) also shows typical features of cold working (for metallographic images see online supplementary material). All of these techniques were commonplace in the steppe objects (Mei, 2003; Qian, 2006).

Tin bronze was the most common alloy found in the Ili region, followed by pure copper (Ling et al., 2008). Other major elements, such as arsenic and antimony, which are often seen in other parts of Xinjiang (e.g., Tianshanbeilu), or leaded bronze in the Central Plains of China, appear to have been far less significant in Ili. The presence and absence of tin (0–15.6%) and arsenic (0–3.5%) divide the entire assemblage into three major groups (Table 2: pure copper, bronze [copper + tin], and arsenic bronze [copper + tin + arsenic]). The highest percentages of tin are found in the mirror (D0347: 15.6%) and two knives (XY0496: 13.5%; D0432: 12.6%). Nevertheless, another mirror was made by pure copper (XY0657), indicating no simple correlation between object typology and alloying technology. Moreover, tin appears more important than arsenic as the number of bronzes is clearly larger than that of arsenic bronzes. This is rather intriguing as arsenic often attracts more attention than tin in Xinjiang archaeometallurgy, as it is often regarded as one of the steppe features (Pollard et al., 2017; Qian, 2006).

**TABLE 2:** Lead isotopic data by MC‐ICP‐MS

Sample name	208/204	2 SD	207/204	2 SD	206/204	2 SD
D0111	38.013	0.007	15.576	0.002	18.199	0.001
D0287–2	37.579	0.017	15.542	0.003	18.047	0.002
XY0075	38.149	0.003	15.602	0.001	18.014	0.000
XY0661	38.478	0.005	15.610	0.001	18.376	0.001
D0078	38.428	0.006	15.609	0.001	18.307	0.001
D432	38.090	0.007	15.578	0.002	18.209	0.002
XY0476	37.891	0.006	15.526	0.002	18.078	0.001
XY0156	38.012	0.006	15.540	0.002	18.269	0.002
XY0151	37.967	0.003	15.548	0.001	18.114	0.001
XY0179	37.911	0.007	15.552	0.002	18.021	0.002
XY0048	38.059	0.004	15.565	0.001	18.250	0.001
XY0226	37.975	0.012	15.541	0.003	18.152	0.002
D0287–1	37.585	0.003	15.529	0.001	18.029	0.000
D0287–3	37.490	0.004	15.498	0.001	18.018	0.000
D0014	37.853	0.009	15.561	0.002	18.261	0.001
XY0059	37.859	0.009	15.521	0.002	18.076	0.001
XY0688	37.937	0.008	15.540	0.002	18.090	0.002
XY0479	38.204	0.004	15.569	0.002	18.437	0.002
XY0496	38.012	0.004	15.560	0.001	18.212	0.001
D0287–4	37.545	0.011	15.538	0.002	18.046	0.002
D0073	38.186	0.008	15.647	0.002	18.326	0.001
XY0148	38.170	0.010	15.584	0.002	18.214	0.001
D0008	37.574	0.003	15.462	0.001	18.041	0.001
D0077	37.575	0.002	15.464	0.001	18.036	0.001
XY0223	38.041	0.004	15.561	0.001	18.183	0.001
Copper ore (Nulasai)	37.760	0.003	15.498	0.000	18.057	0.000
D0083	37.968	0.004	15.558	0.001	18.243	0.001
D0010	37.891	0.022	15.525	0.003	18.080	0.002
D0280	37.886	0.002	15.553	0.001	18.250	0.001
D0069	38.049	0.003	15.573	0.001	18.209	0.001
D0080	37.963	0.004	15.563	0.001	18.404	0.001
D0287–5	38.021	0.004	15.573	0.001	18.168	0.002
XY0082	38.485	0.006	15.618	0.002	18.345	0.002
D0347	38.146	0.005	15.592	0.001	18.301	0.001
D0075	38.088	0.007	15.560	0.003	18.099	0.003
XY0657	37.855	0.009	15.521	0.003	18.132	0.002
D0109	38.016	0.009	15.539	0.003	18.037	0.003
XY0049	38.707	0.006	15.657	0.001	18.521	0.001
XY0150	37.824	0.003	15.552	0.001	18.100	0.001

The range of lead isotopic ratios falls in the range of common lead (^206^Pb/^204^Pb: 18.04–18.52 ^207^Pb/^204^Pb: 15.46–15.65, ^208^Pb/^204^Pb: 37.49–38.71) and illustrates no distinctive grouping patterns. This continuous dataset implies that this group of objects were made by copper from a limited number of sources with similar geological history or perhaps mixing and recycling of objects obscure the original boundaries in the data (Table 2).

A more diversified picture is found within the trace element data. Cobalt, nickel, arsenic, and gold are usually below 500–600 μg/g. This corresponds to the low results on arsenic by SEM‐EDS, most of which are reported below detect limit (Table 3). The concentration of antimony in the majority of objects are reported below detect limit of ICP‐AES, but a few show obviously higher amounts between 1,000–10,000 μg/g (e.g., XY0150: 9523.1 μg/g). Selenium, tellurium, silver, and bismuth show concentration around thousands to tens of thousands μg/g. Unfortunately, very few copper‐based objects in Xinjiang have been analysed with this exhausted list of chemical elements, so it is difficult to draw reference from other datasets, particularly for selenium and tellurium. Silver and bismuth are occasionally published in eastern Xinjiang, but their concentrations are much lower than these of Ili (Liu et al., 2020). It is also noticeable that the concentration of lead is usually below 1%, with one exception containing lead around 5% (XY0049). Sample XY0688 shows unexpectedly high amounts of bismuth and tellurium (around 30, 000 μg/g), which might be due to its extremely heavy corrosion.

**TABLE 3:** Alloying data produced by SEM‐EDS

Sample number	Object name	Elements (Wt%)				
		Cu	Sn	As	Others	Major elements
D0109	Knife	97.4	2.7	—	—	Cu‐Sn
D0014	Arrowhead	97.1	2.9	—	—	Cu‐Sn
D0347	Mirror	80.0	15.6	3.5	S:1.0	Cu‐Sn‐as
D0280	Hairpin	94.6	5.4	—	—	Cu‐Sn
D0012	Chisel	65.7	9.5	—	O:24.8	Cu‐Sn (heavy corrosion)
D0008	Spearhead	96.6	3.4	—	—	Cu‐Sn
D0073	Bowl	77.1	—	—	O:22.9	Cu (heavy corrosion)
D0075	Knife	92.1	7.9	—	—	Cu‐Sn
D0080	Bowl	95.1	5.0	—	—	Cu‐Sn
D0111	Belt buckle	70.9	—	—	O:17.2 Cl:12.0	Cu (heavy corrosion)
D0287	Arrowhead	90.7	9.3	—	—	Cu‐Sn (heavy corrosion)
D0432	Knife	87.4	12.6	—	—	Cu‐Sn (Pb)
D0010	Cauldron	97.8	2.2	—	—	Cu‐Sn
D0077	Cauldron	100.0	—	—	—	Cu
XY0657	Mirror	83.2	—	—	O:15.5 Cl:1.3	Cu (heavy corrosion)
XY0496	Knife	86.50	13.5	—	—	Cu‐Sn

## DISCUSSION

### Pure copper technology of the cauldrons

The cauldrons, which are also known as the Fu vessels in Chinese literature, formed an important Eurasian phenomenon in antiquity. Similar types of objects have been found spread from the Danube River to Northeast part of China, with over 50 cauldrons found in Xinjiang. However, most of these were chance findings and are best dated according to their typology. Scholars categorise the cauldrons from Xinjiang into three major types depending upon the handle styles, positions and number, and upon the style of the foot (ring foot or tripod) (Ma, 2017; Mei et al., 2005). The chief characteristic of Type I is the two straight handles standing on the edge of the mouth (e.g., XY0150, XY0151), which distinguishes itself from Type II, which has two oblique handles inserted just below the edge of the mouth (e.g., D0077, XY0479, XY0059, XY0476 and XY0478). Ring foot is common in both Type I and II. So far, only four cauldrons in Type III are known to scholars, all of which were discovered around Ili. Being different from Type I and II, Type III shows four handles (two oblique and two vertical) and three independent legs (e.g., XY0477 and XY1048). The earliest cauldron with secure excavation context is the piece of Type I at Lanzhouwanzi. The radiocarbon date from the associated layer suggests a chronology of around 1,665–1,452 BC (68.3%, 1745–1,415 BC, 95.4%) (Zhang & Zhao, 1991). However, quite a few publications have misused the uncalibrated radiocarbon result and argued for a period equivalent to the late Shang dynasty (ca. 1,250–1,045 BC). But the association between this radiocarbon date and the circulation of Type I cauldron is suspicious. If the radiocarbon result holds true, Type I appears more likely to start at least 200 years earlier than late Shang in the Central Plains and gradually disappear around the period equivalent to the Warring States (ca. 475–221 BC). The mistaken use of radiocarbon dates could also be encountered in other cases in Xinjiang copper objects as well. In general, Type II is geographically restricted to western Xinjiang, mainly the Ili and Altai region. Its circulation appears in a similar length but slightly later period than that of Type I, ranging from 10th century BC to the rise of the Han dynasty. Type III can be dated to seventh century BC–second Century AD by the co‐exited tomb contents (e.g., bronzes and funeral figurines; Mei et al., 2005).

The chronology of cauldrons should be treated with more caution. Although these types of cauldrons display distinctive typological features, it is still impossible to tell the precise chronology of their production, circulation, and deposition. The same issue applies to many other types of copper‐based objects discovered in Xinjiang. As mentioned above, this is ultimately due to the fact that many of them are chance findings therefore lack proper archaeological context to be radiocarbon dated. Meanwhile, only a limited number of key sites in Xinjiang have been published with radiocarbon dates with full stratigraphic information (e.g., Jia et al., 2017; Tong et al., 2020). Objects outside these sites can be only loosely dated by their typology or associated objects in the same context/archaeological culture.

So far, the Ili region has yielded the largest number of the cauldrons found in China (28 out of 51 in total). It is often assumed to have been the most important centre for the production of cauldrons. Intriguingly, all nine cauldrons analysed in the current study are made from pure copper. This differs from the dominant alloying technologies in local and neighbouring regions, such as the tin or arsenic copper/bronze in Xinjiang and Siberia (Hsu et al., 2016; Mei, 2000; Qian, 2006) or leaded bronze in Central China. This was noted by Mei et al. (2005). Given the wide distribution of cauldrons across Eurasia, they argue that the choice of pure copper for this special type of object could be linked to southern Siberia, where more cauldrons from the same period have been confirmed as pure copper. The growing body of new analyses further supports pure copper as the dominant material for cauldrons from Xinjiang.

The technological choice of the Ili cauldrons raises a set of new and intriguing questions concerning the ways in which local metallurgists overcome the challenge of melting down pure copper with the melting point up to 1,083°C. Moreover, cooling of pure copper may result in many shrinkage cracks. How was this specific recipe of casting was determined for cauldrons whilst a range of other choices were certainly known by the craftspeople? Some speculations could be provided in the light of the lifestyle of pastoralism in a broader context. Cauldrons are very likely to be multifunctional on the steppe. As evidenced by the rock art (Chen, 2008), at least some specific types of cauldrons associated with not only daily life but also ritual ceremonies. The decorative patterns of cauldrons are however much simpler than the bronze ritual vessels in Shang and Zhou dynasties of the Central Plains. There is no such need as casting ritual vessels in Shang and Zhou to reduce the melting point of the alloy by addition of tin and/or lead to copper allows the metal to stay in liquid long enough to fulfil the complicated moulds. Making these cauldrons requires much less complicated alloying technologies. It might be possible that the cauldrons made by pure copper may exhibit specific properties such as more red colour or low hardness that could be useful in their daily use or ritual ceremonies. Second, high mobility of the steppe lifestyle poses extra challenges to maintain the stable supply of tin for the routine metallurgical practice, especially for the objects in larger sizes. This is primarily because that tin sources are much rarer than copper.

**FIGURE 3:**
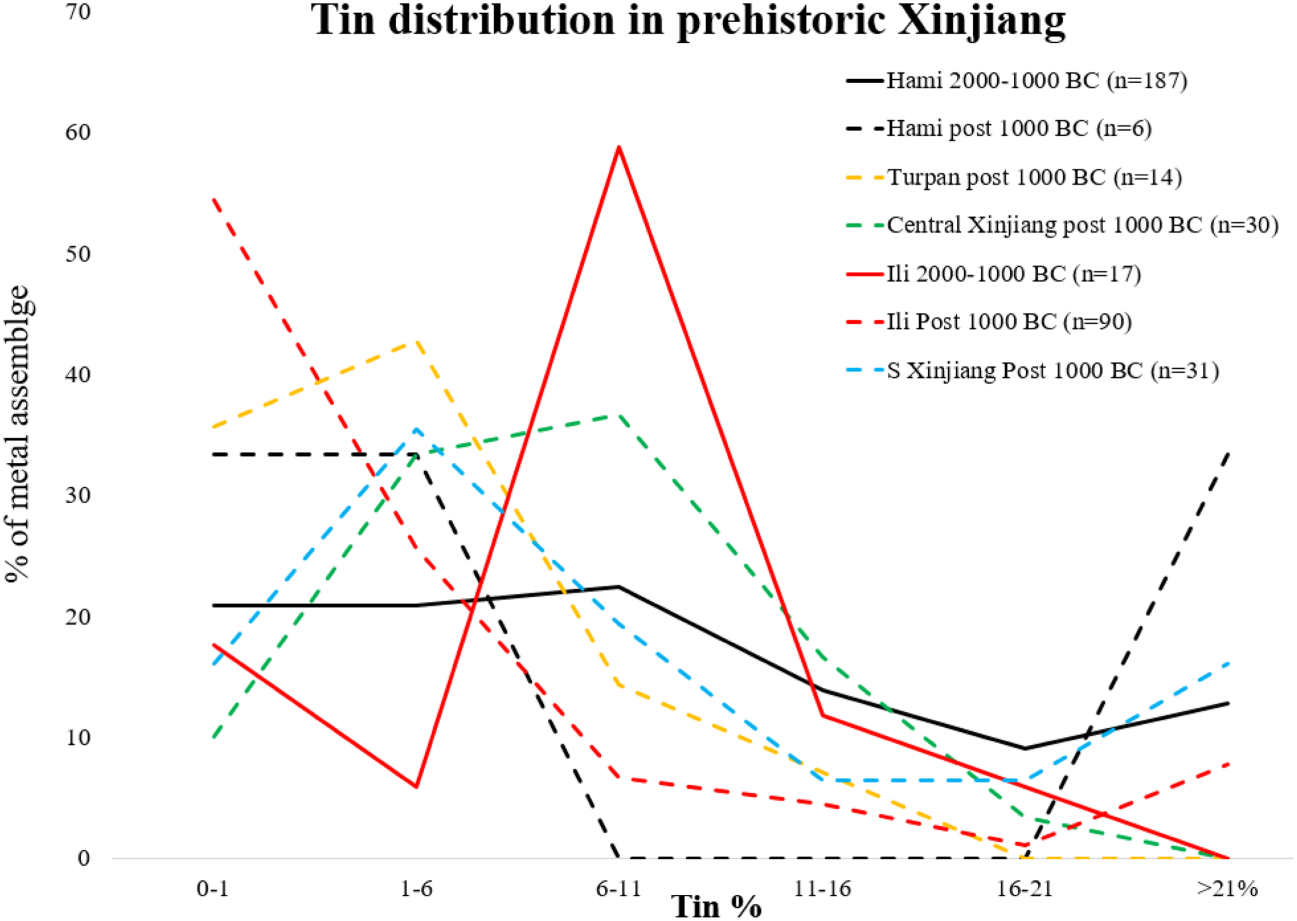
Distribution of tin in prehistoric Xinjiang [Color figure can be viewed at wileyonlinelibrary.com]

The increasing dependence on pure copper can be seen in not only cauldrons at Ili but also various types of objects across Xinjiang during the early Iron Age. Figure 3 summarises the changes in the alloying technologies in different parts of Xinjiang. During the Bronze Age, over 70% of the overall metal assemblage in Ili (including tools, weapons and ornaments, n = 17) contains tin in the range of 6–16%, with approximately 60% falling in the range of 6–11%. The contemporary metal objects from Hami are also tin bronze. Around 15% of the Hami assemblage (n = 187) contains a tin concentration of over 21% and over 80% contain tin over 1%. This supports the idea that both Hami and Ili during the second millennium BC were able to produce a large number of tin bronzes. However, the first millennium BC witnessed a radical shift in the supply of tin in both regions. In Ili, the tin concentration of nearly 80% of the metal objects falls below 6%, and over half of the metal objects contain less than 1% tin. Similar patterns can be also found in the eastern part of Xinjiang, such as at Hami and Turpan. In fact, with the exception of Central Xinjiang represented mainly by the site of Shimenzi at Hutubi Changji (Wang et al., 2014), all other major regions (Ili, Hami, Turpan, South Xinjiang) show a broad decrease in the use of tin in the early Iron Age. Although more research is necessary to investigate the underlying causes of this change, it is reasonable to say that the pure copper technology for cauldrons was not an isolated case in a broader archaeological context. Intriguingly, in different parts of Eurasia such as the Iranian plateau, east Mediterranean Sea, Caucasus, and the Central Plains of China, local production of metal saw increasing emphasis in tin and bronzes, even though many of these regions already stepped in early Iron Age (Cuénod et al., 2015; Ho & Erb‐Satullo, 2021). The decline of tin in Xinjiang around the late second to early first millennium BC appears rather distinctive.

**FIGURE 4:**
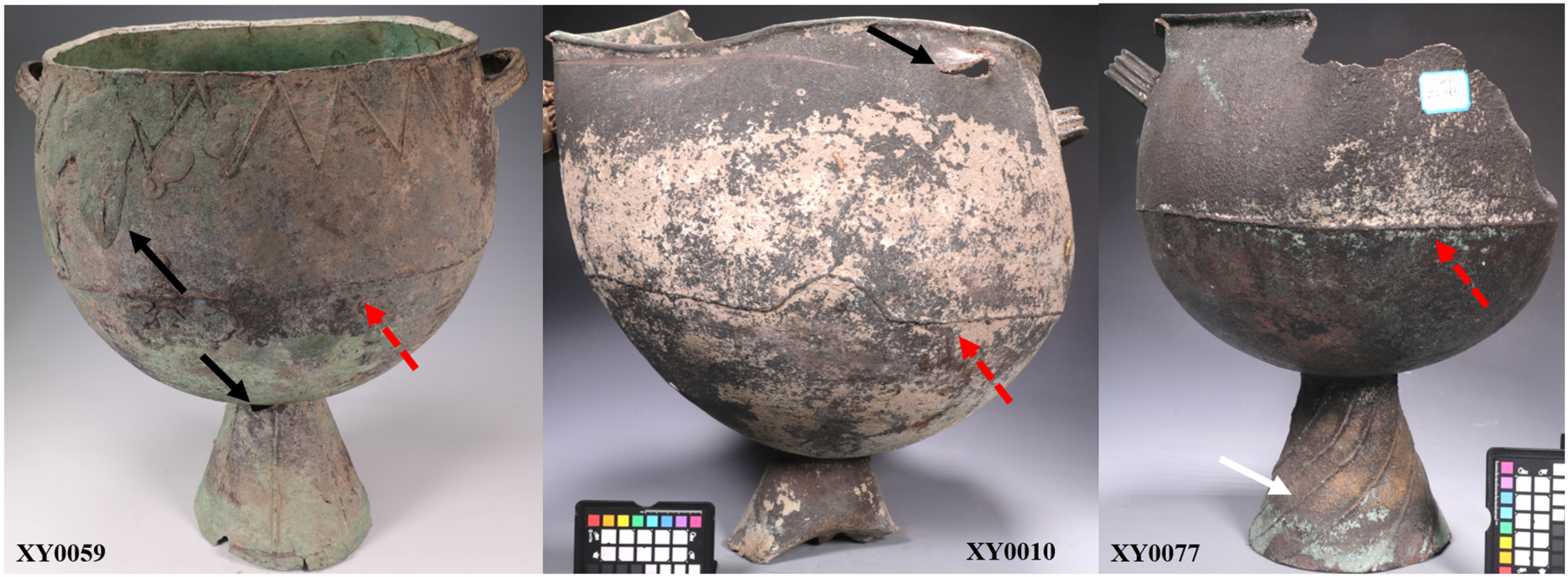
Casting features on cauldrons *Notes*: Black arrow: repairing and casting defects; white arrow: decorative lines; dash red arrow: decorative or moulding lines) [Color figure can be viewed at wileyonlinelibrary.com]

**FIGURE 5:**
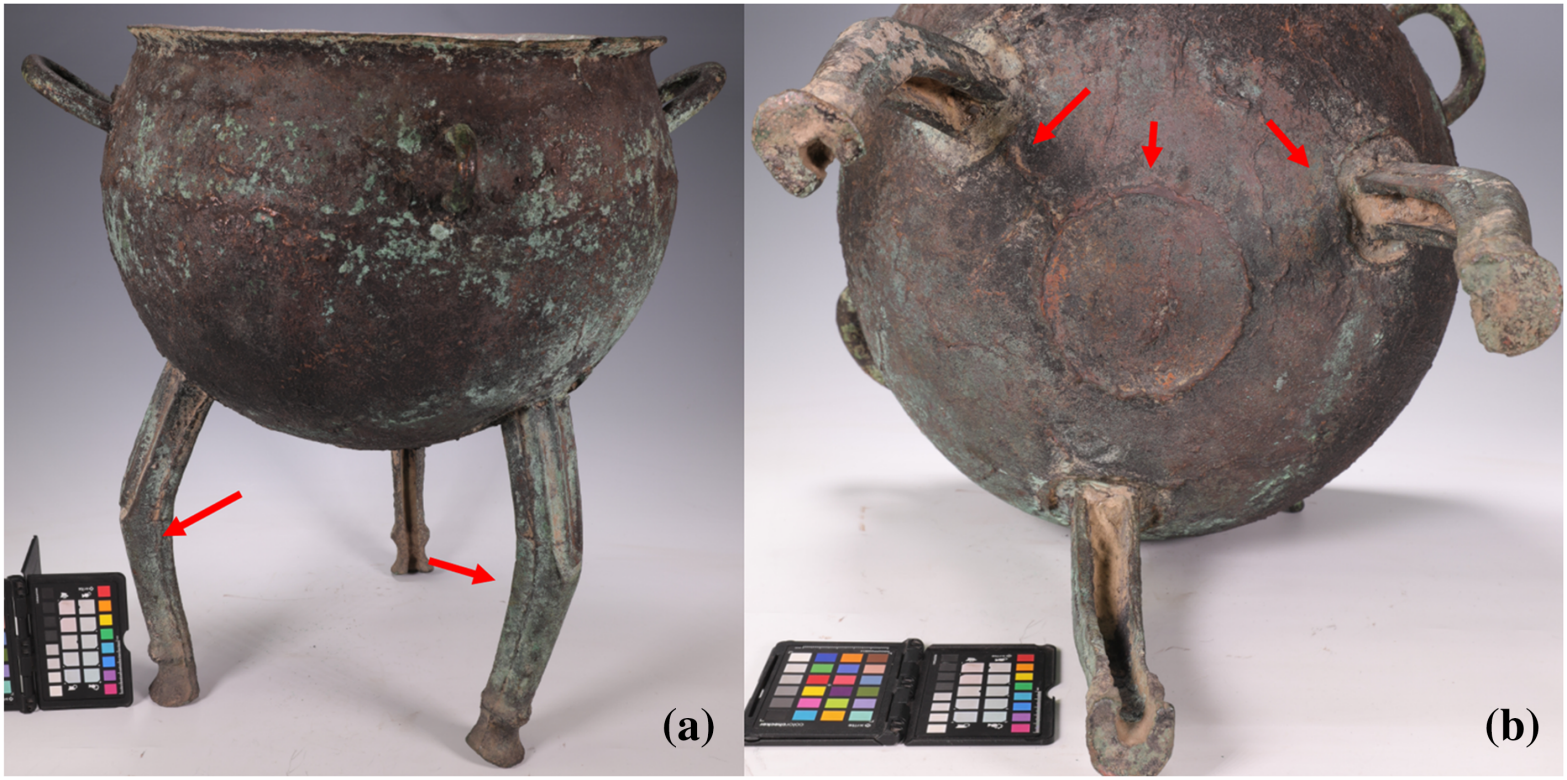
Moulding lines on the cauldron XY0477 (solid red arrows) [Color figure can be viewed at wileyonlinelibrary.com]

In addition to alloying technology, it is also important to remember that rather than being forged, at least some of the cauldrons from around Xinjiang were made by piece‐mould casting, which was invented in Central China. Scholars have noted that the horizontal line often observed on the upper body of a cauldron could represent the moulding line (the line where two pieces of mould meet) of the piece mould casting (Hide, 1994; Mei et al., 2005). This becomes more complicated because these lines could also be part of decoration (Figure 4). The curved pattern on XY0010 implies the horizontal line is more likely created for decorative purpose. It is more obvious in the case of the curved lines on the ring foot of XY0077, which are by no means moulding lines. More robust evidence for piece‐mould casting on the Ili cauldrons comes from XY0477 (Figure 5). Clear moulding lines can be observed from the side surface of the three legs as well as the bottom of the object. The round line in Figure 5b is a result of assembling three moulds together with one core in the middle, which is widely used for creation of the bronze ritual vessels in Central Plains of Eastern Zhou (ca. 770–256 BC). The casting defect together with repairing on these cauldrons echoes the difficulty in choosing pure copper to cast large vessels as well as the long and complex life history of these objects (repairings on Figure 4, more photos in online supplementary material). Some cauldrons in Ili probably illustrate the western boundary of the technological sphere of piece‐mould casting originated in the Central Plains. It is still necessary to bear in mind that others display no solid evidence such as moulding lines that allows us to associate their casting technique with piece moulds. More research is needed to combine compositional analysis with X‐ray photography and metallography to explore the complex technological traditions exhibited by these cauldrons.

### Mixing of different types of copper at Ili

This study is limited by the small number of trace elemental datasets for prehistoric metal objects in Xinjiang. Datasets are only available for the metal assemblage in Ili (Table 4) and the Tianshanbeilu Cemetery at Hami (Liu et al., 2020). However, a comparison of these two datasets is useful.

**TABLE 4:** Trace elemental data by ICP‐AES (bdl, below detection limit)

Lab number	Object type	Pb %	Element (μg/g)								
			Co	Ni	As	Sb	Se	Te	Ag	Au	Bi
XY0496	Knife	0.1628	1.3	bdl	47.3	bdl	1694.9	2989.3	4306.5	bdl	3524.3
XY0476	Cauldron	0.1403	6.8	bdl	241.4	bdl	1750.7	4275.4	15025.3	bdl	6483.0
D0280	Hairpin	0.1280	0.6	17.2	72.0	1710.4	135.0	1309.5	3525.9	502.3	1709.7
XY0150	Cauldron	0.0589	3.5	10.0	279.3	9523.1	1023.6	1966.9	3663.6	bdl	3563.7
D0069	Hairpin	1.0443	8.7	bdl	479.3	bdl	3503.7	9757.3	4213.3	bdl	13402.5
XY0151	Cauldron	0.0902	6.3	1.9	136.8	bdl	936.5	2423.2	24787.5	bdl	5334.4
XY0059	Cauldron	0.1116	3.2	bdl	164.7	bdl	292.1	4518.4	23841.7	bdl	4254.6
XY0072	Sickle	0.0152	0.7	bdl	4.5	bdl	116.0	1704.4	3850.0	bdl	2212.0
D0010	Cauldron	0.8705	4.5	6.7	119.9	423.5	233.3	1682.0	10110.4	bdl	2046.7
XY477	Cauldron	0.0206	5.1	99.9	83.4	bdl	703.9	3888.2	4299.0	bdl	4184.9
XY0152	Cauldron	0.0126	0.6	12.6	10.7	bdl	268.0	1784.5	1683.1	bdl	1993.6
XY0049	Figurine	5.1713	11.1	37.9	353.9	5831.0	1401.3	4977.6	7847.9	bdl	6098.0
XY0156	Plate	0.0228	0.8	15.5	16.9	bdl	199.0	1471.9	2129.1	bdl	1866.5
XY0688	Pot	0.2538	34.1	334.1	694.1	bdl	0.0	33781.2	8676.5	bdl	27680.0
XY0479	Cauldron	NA	0.4	12.6	12.7	bdl	150.6	973.0	1564.1	bdl	1295.2
D0110	Sickle	NA	0.2	0.0	1.4	bdl	196.5	1080.6	2871.0	2.4	1307.7
XY0478	Cauldron	NA	0.2	28.5	15.5	bdl	199.1	1334.8	2224.7	0.4	1739.7
D0077	Cauldron	NA	1.3	4.4	250.9	469.5	149.0	1052.8	1401.3	bdl	1382.4

An initial characterisation using the Oxford system, which characterises copper according to the presence (> 0.1%) or absence (< 0.1%) of arsenic, antimony, silver, and nickel (Bray et al., 2015; Pollard et al., 2017, 2018). Tianshanbeilu shows multiple groups of copper, featured by the presence of arsenic, antimony, and silver. Liu et al. (2020) point to fahlore (tennantite‐trahedrite) as the major source of copper for the Tianshanbeilu metal based on the strong correlation between arsenic and antimony. The impurity pattern in the Ili metal assemblage is remarkably different. Over 80% of the metal objects correlate with just one copper group, (CG4, silver‐only), followed by CG7 (Sb‐Ag, merely 17%). This shows that in comparison to Tianshanbeilu, Ili probably had to access a smaller number of copper sources (Table 5).

**TABLE 5:** Distribution of copper groups for the metal assemblage in Ili and Hami Tianshanbeilu

Copper group site	CG1 pure copper	CG3 Sb‐only	CG4 ag‐only	CG7 Sb‐ag	CG9 as‐ag	CG12 as‐Sb‐ag
Ili (n = 13)	0	0	83%	17%	0	0
Tianshanbeilu (n = 16)	27%	4%	23%	14%	23%	9%

*Note*: For definition of copper groups see Bray et al., 2015.

However, a single copper group does not necessarily establish only one single source of copper (Liu et al., 2019). Multiple sources of copper are indicated by the lead isotopic data. A change in the source of copper in Ili during the transition between the Bronze Age and early Iron Age is illustrated in Wang et al. (2018). The new lead isotope ratios make a further contribution to the overall data available and supports observation made by Wang and colleagues. A much wider comparative study of the lead isotopic data of objects, and modern and ancient copper/lead ores have been carried out to explore the possible sources of copper for Ili. We follow the geographical framework set up in Hsu and Sabatini (2019) and divide Xinjiang into six major regions, namely North Xinjiang (the Altai Mountain), South Xinjiang (the Kunlun Mountain), South Xinjiang (the Tarim Basin), and East/South/West Tianshan Mountain, with the ancient copper mining/smelting site Nulasai being highlighted for further analysis. The basic dataset is also updated by adding more lead isotope ratios of copper and lead ores in the Ili region and the removal of the gold data from the original dataset compiled by Hsu and Sabatini (2019).

**FIGURE 6:**
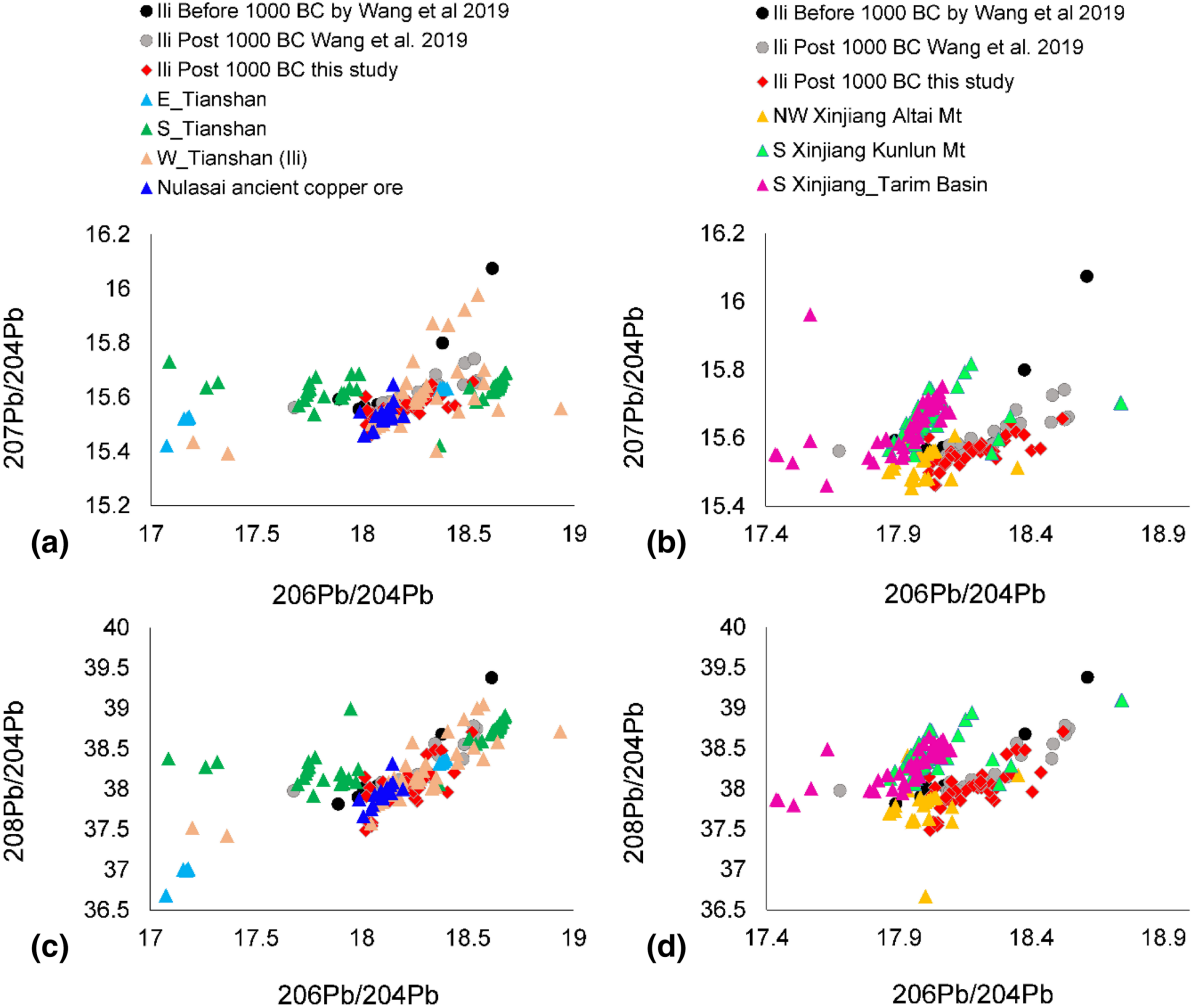
Comparison of lead isotopes between Ili and potential copper sources [Color figure can be viewed at wileyonlinelibrary.com]

Lead isotope data in Figure 6(c‐d) rule out South Xinjiang (both Kunlun and Tarim) as a possible source for Ili's copper as they show much higher values in ^207^Pb/^204^Pb and ^208^Pb/^204^Pb than those of the Ili objects. Although the lead isotopic data for the Altai Mountains in North Xinjiang appears to overlap with the lower part of the Ili data (^206^Pb/^204^Pb ≈ 18.0), when compared with the Tianshan Mountain, it is a much less likely source of copper for Ili. The western part of Tianshan shows the best match with the Ili objects. Previously there was insufficient data available to characterize the copper mines in the area of the East Tianshan. The South Tianshan shows a more diverse distribution of lead isotopes, but interestingly, none of these data points fall into the distribution of Ili objects.

The lead isotope data of the ancient copper mine at Nulasai in Ili has been highlighted in Figure 6(a‐b). On one hand, the clear overlap between Nulasai and the Ili assemblage implies that at least some of the objects could be made from the Nulasai copper. On the other hand, the range of lead isotope data of the Ili metal objects extends beyond that of Nulasai. This is either because the current dataset does not capture the overall heterogeneity of the Nulasai mine, or that copper from other mines with different lead isotopes were involved. The latter appears more likely as various types of ores (malachite, galena and chalcocite) show similar lead isotopic signature. This also helps to explain the mismatch in the alloying data. Although pure copper was possibly produced by Nulasai, its primary product was actually arsenic copper (Li & Mei, 2001; Mei, 2003). But the local objects at Ili, such as the cauldrons, were mainly tin bronze and pure copper.

**FIGURE 7:**
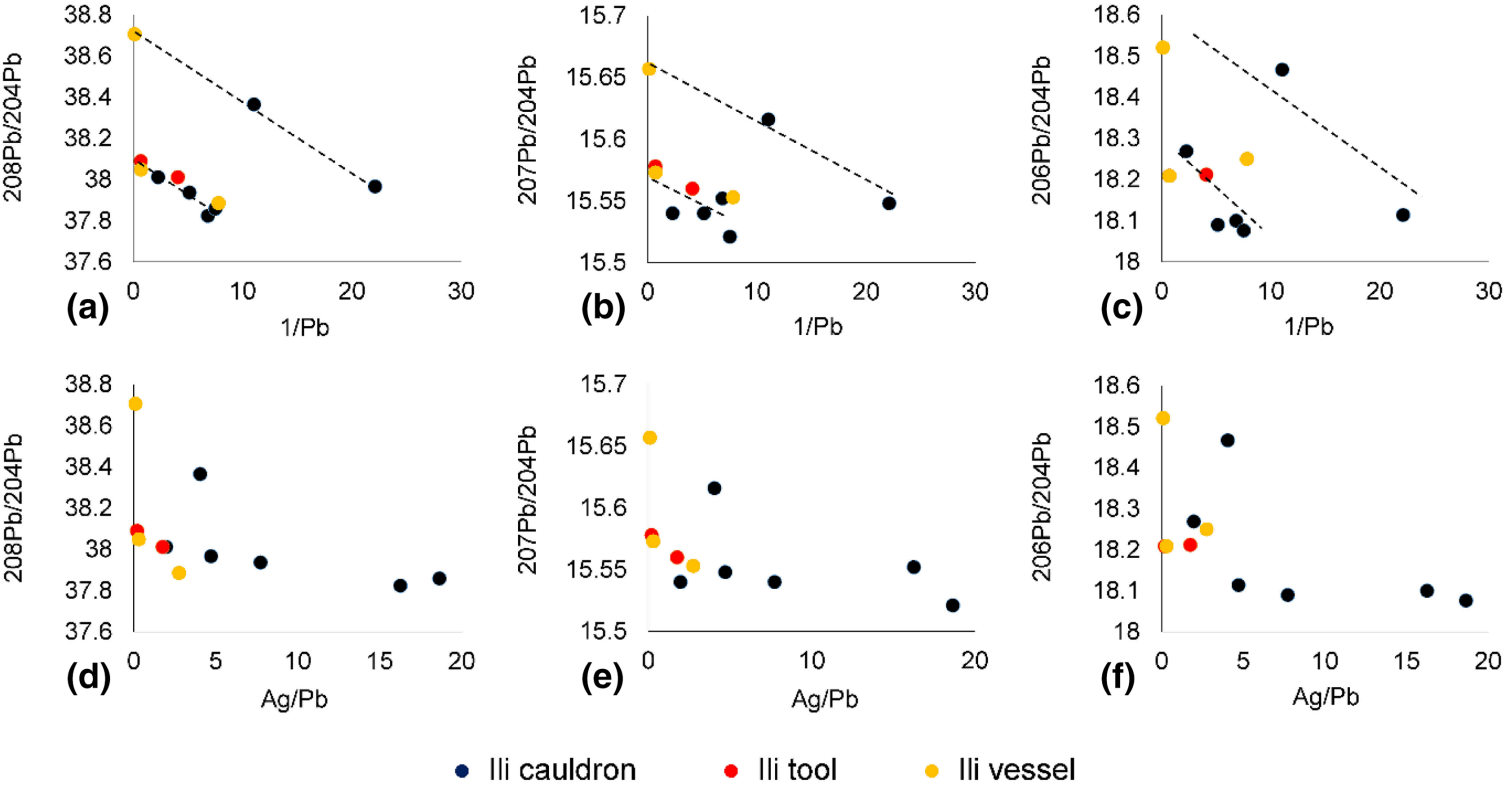
Mixing of two types of copper for the Ili metal objects *Note*: In a–c dash lines are for illustrative purpose to highlight the linear regression resulted from mixing [Color figure can be viewed at wileyonlinelibrary.com]

Further clues can be found by combining lead isotope data with lead concentration and trace elements. Figure 7(a‐c) discovers at least two linear regressions by plotting lead isotope data against 1/Pb(%), which can be mathematically proven as a result of mixing two sources of copper (see Liu 2016: Appendix III for relevant mathematics). The amount of silver is further added to Figure 7(d‐f). The overall data structure of linear trend remains the same, suggesting that the parental sources can be not only characterised by lead isotope ratios but also non‐argentiferous (associated with higher lead isotope ratios) and argentiferous (associated with lower lead isotopes) copper. It is also interesting to note that the practice of mixing and recycling can be applied to all types of objects in Ili. This carries deeper social implication if one compares with the management strategy of metal at the last capital of the Shang dynasty, Anyang (ca. 1,200–1,050 BC). Social hierarchy performed a major part in the metal circulation at Anyang, where top‐elite bronze objects were made by targeted alloying recipes and well‐refined, non‐recycled metal. The recycled metal objects are more likely to be recovered from lower elite tombs (Liu et al., 2020). In Ili, mixing and recycling is more widely applied to all sorts of metal objects. Although there is no doubt that steppe societies were also structured with highly complex social hierarchies, from the perspective of raw metal resources, it takes a rather different form from the Central Plains. More emphasis is probably put on gold, silver, and sophisticated decorations of the objects rather than the underlying raw material (Rawson, 2017), which might still due to their mobile life styles.

## CONCLUSIONS

The new chemical and isotope analysis of the copper‐based objects in the early Iron Age Ili region provides new insights into the metallurgical technology and local strategy of metal management in the steppe context. The cauldrons discovered at Ili demonstrate not only a new object type but also a different choice of alloying practice (pure copper) and casting technique (piece‐mould casting). The decline in the supply of tin during the early Iron Age can be also seen from other types of objects across Xinjiang. For the first time, a clear pattern indicative of mixing various types of copper can be illustrated for the prehistorical metal production in Xinjiang, demonstrating the great potential of combining lead isotopes, trace elemental, and alloying data. As a crucial copper source at Ili, the Nulasai site corresponds to the less argentiferous one with lower lead isotopic values. At least another source of copper remains to be identified in the future.

### PEER REVIEW

The peer review history for this article is available at https://publons.com/publon/10.1111/arcm.12770.

## Supporting information




**Data S1.** Supporting Information

## Data Availability

All the data is available in the paper.
