# Supplementary material for: New scientific analyses reveal mixing of copper sources in the early Iron Age metal production at Ili, western China
Source: Archaeometry. 2022 Mar 15;64(Suppl 1):98–115. doi: 10.1111/arcm.12770 (PMC9314118; doi:10.1111/arcm.12770)
Supplement: Supplementary file 1 — Data S1. Supporting Information [file ARCM-64-98-s001.docx]

Metallographic analysis of Ili metal objects

| 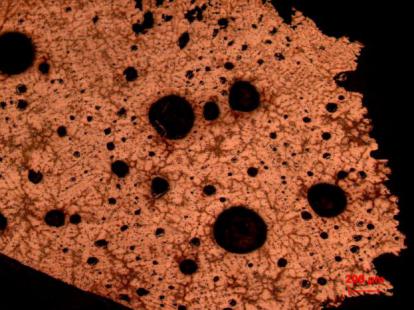  **D0077 (Cauldron)** | 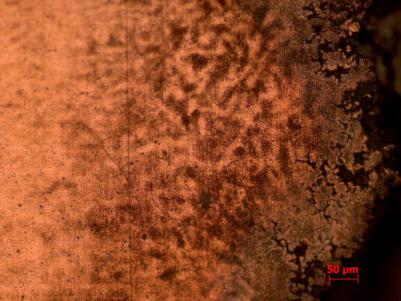  **D0280 (Hairpin)** |
| --- | --- |
| 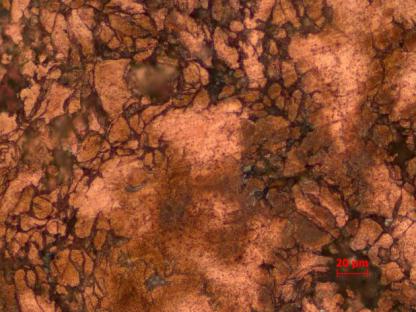  **D0075 (Knife)** | 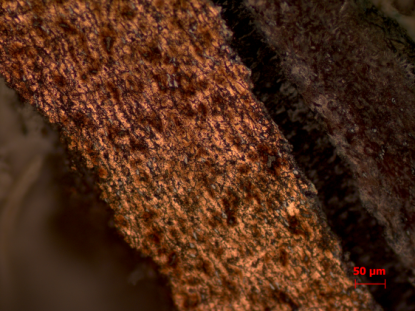  **D0432 (Knife)** |
| **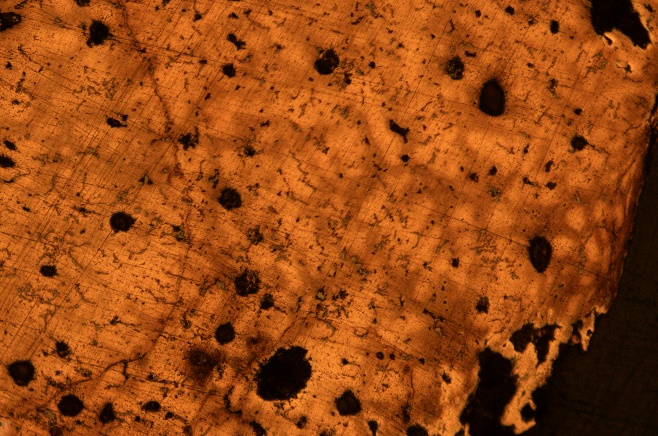**  **XY0688 (Pot)** | 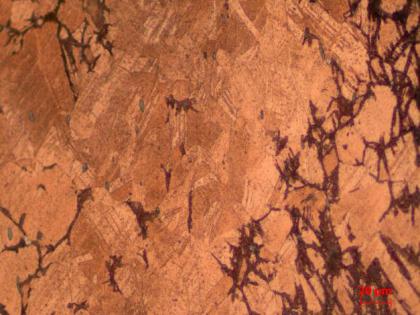  **D0008 (Spearhead)** |
| 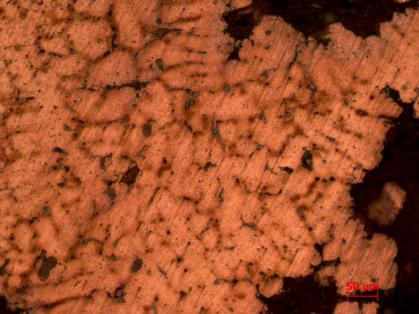  **D0010 (Cauldron)** |  |

Note: Clear dendro-structures are revealed by the metallographic analyses, demonstrating casting as the most important technology for these objects. Further cold-working is indicated by the slip lines in the knife (D0432). D0008 shows twin grains by annealing. Either casting or cold-working is commonly encountered in the steppe metal-working.


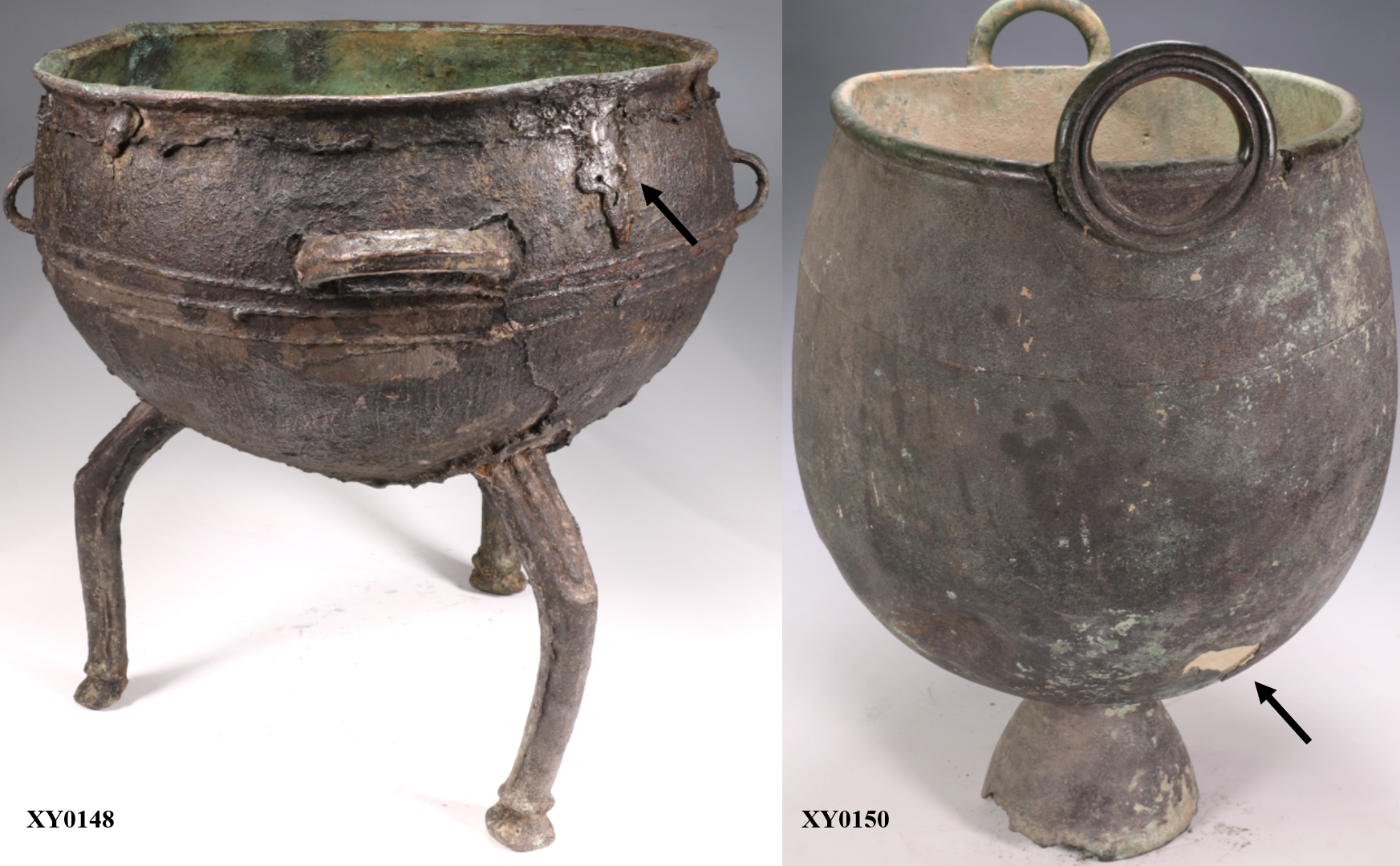


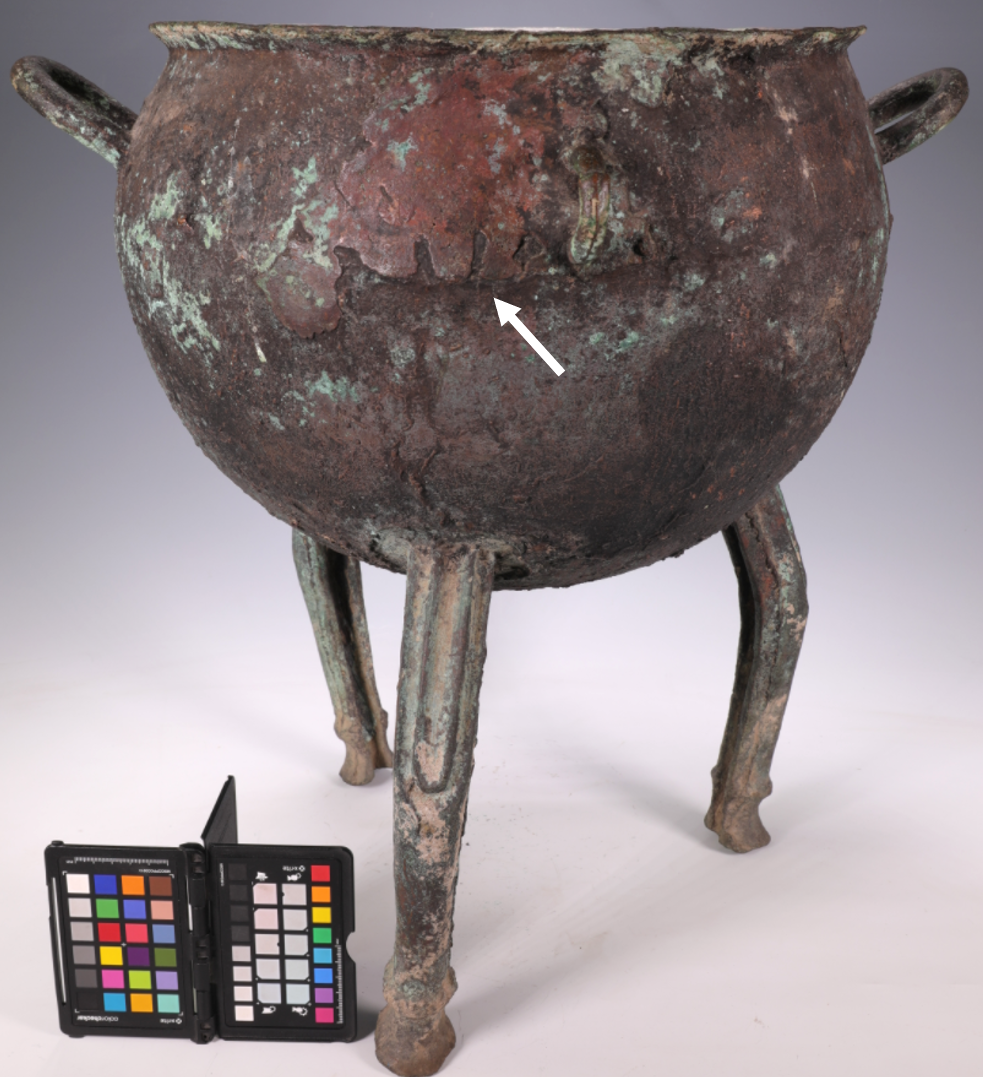


Casting Defects and repairing of cauldrons
